# Decreased circulating levels of free triiodothyronine in Sepsis children and correlation analysis

**DOI:** 10.1186/s12887-022-03756-7

**Published:** 2022-11-29

**Authors:** Li’na Fu, Wenjun Long, Tonglin Liu, Yu Chen, Shimin Wu, Dandan Li, Kang Liu, Yuan Gao, Xiaoping Luo

**Affiliations:** 1 Department of Pediatrics, Tongji Hospital, Huazhong University of Science and Technology, Wuhan, 430000 China; 2 Department of Pediatric Surgery, Tongji Hospital, Huazhong University of Science and Technology, Wuhan, 430000 China; 3 School of Public Health, Guangzhou Medical University, Guangzhou, 511436 China

**Keywords:** Free triiodothyronine, Sepsis, Children, Inflammatory biomarkers

## Abstract

**Background:**

Intensive physical stress in sepsis can induce the disorder of endocrine function and impact the clinical course and prognosis. Low T3 syndrome has been verified to be the predictive indicator of poor prognosis in several researches. Reports on the influence factors of thyroid hormonal levels in children with severe sepsis are rare. We aim to investigate the thyroid hormonal variations in the course of sepsis and analyze that how to be affected by clinical data and inflammatory biomarkers.

**Methods:**

In the case-control study, 184 children with sepsis and 323 controls were included in Tongji Hospital, Wuhan, China, in 2019. Data on clinical and inflammatory parameters were collected from all participants. Circulating FT3(Free Triiodothyronine) levels were measured by Electrochemiluminescence immunoassay. Finally, we investigated the correlation between FT3 and related variables with linear regression analysis.

**Results:**

Serum FT3 was lower in the sepsis group than in control group(2.59 + 1.17 vs 2.83 + 1.01 pg/mL, *p* < 0.05). Significant moderately negative correlations(**|**r**|** > 0.3) of FT3 levels with ferritin, PCT, duration of symptoms, SOFA score, and mortality were revealed. Moreover, we observed that FT3 had the positive correlation with albumin, as well as white blood cell count.

**Conclusions:**

Concentrations of serum FT3 are dramatically declined in sepsis children than in control children. Our results demonstrate that recognizing the potential abnormality of thyroid hormones in sepsis patients and examine timely through abnormal common clinical data and inflammatory biomarkers is a fine option.

## Background

Sepsis is a major cause of children in the world with high morbidity, high mortality, and high expense of medical resources. Globally, patients suffering from sepsis account for 8% in Pediatric Intensive Care Unit (PICU) in high-income countries [1], with the mortality rate fluctuating between 4 and 50%, which depends on the severity of disease, risk factors, and geographical location [2–4]. According to the latest guideline, sepsis is defined as “Life-threatening organ function dysfunction caused by a dysregulated host response to infection” [5] . It has been verified that intensive physical stress in sepsis can induce the disorder of endocrine function, and impact the clinical course and prognosis. The HPT(Hypothalamus Pituitary Thyroid) axis of critical patients is in disorder, and low T3 syndrome is most common among the hormonal changes [6]. Adults or children, low T3 syndrome has been confirmed to be the predictive indicator of poor prognosis in several researches [7–10].

As far as I know, reports on the influence factors of thyroid hormonal levels in children with severe sepsis are rare. Thereby, we aim to investigate the thyroid hormonal variations in the course of sepsis and analyze that how to be affected by clinical indexes and inflammatory biomarkers. By these, we could inspect targeted abnormal thyroid function so as to intervene in time, and probably improve the prognosis of patients with severe sepsis to some extent.

## Methods

### Patients

**Fig. 1:**
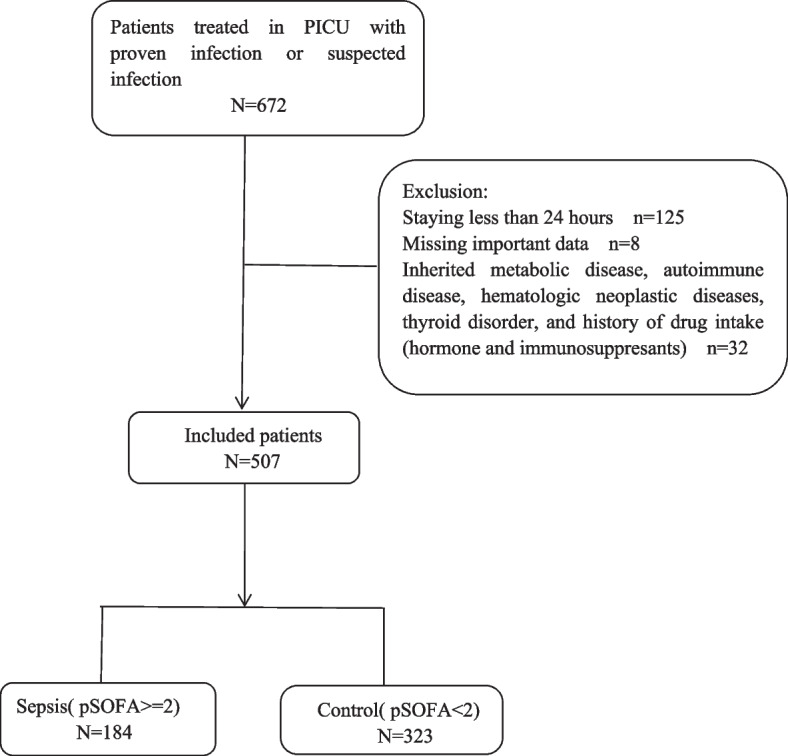
Flow chart of the study pSOFA (pediatric Sequential Organ Failure Assessment)

We respectively performed a single center case-control study of individuals greater than 1 month to 16 years old admitted to PICU in Tongji Hospital affiliated to Huazhong University of Science and Technology from 1st January 2019 to 31th December 2019. A total of 672 patients with suspected or proven infectious diseases were incorporated into the study among 972 individuals. The exclusion criteria were as follows: 1) Staying in PICU less than 24 hours; 2) History of thyroid diseases; 3) Missing important data unable to group; 4) Combined with non-infectious diseases, such as neoplastic diseases, autoimmune diseases, and metabolic disorders. Finally, 507 cases were incorporated into study according to the exclusion criteria, and divided into 2 groups(sepsis group and control group). The diagnostic standard of sepsis referred to the Sepsis-3.0 Definitions in critically ill children [11]. (Referring to Fig. 1).

### Clinical data collection

The following clinical data of all participants within 1 hour admitted to PICU were collected: age, gender, source, duration of symptoms, medical history, Glasgow Coma Sale (GCS), body temperature, respiratory rate, heart rate, blood pressure, and fraction of inspired oxygen. The subsequent data of patients were collected in the duration of therapy in PICU, including type and dose of cardio-active drug, invasive ventilator therapy, length of staying in PICU, and treatment outcome. Sources of patients were classified into 3 categories: general ward, pediatric emergency, and junior hospital. Mean arterial pressure (MAP) was calculated as (systolic pressure + 2 × diastolic pressure)/3. The SOFA score was calculated according to the literature [11].

### Laboratory measurement

Peripheral arterial and venous blood samples were collected within 2 hours of admission and measured in the Department of Clinical Laboratory (Tongji Hospital, Huazhong University of Science and Technology). Thyroid hormones levels (Free Triiodothyronine, FT3; Free Thyroxine, FT4; Thyroid Stimulating Hormone, TSH) were measured by Electrochemiluminescence immunoassay(Roche Diagnostics，Mannheim, Germany). Ferritin was determined by Particle enhanced inmunoturbidimetric assay(Roche Diagnostics，Mannheim, Germany). White blood cells and platelets were assayed using Automatic blood routine analysis (Sysmex, XE-5000，Japan). Interleukin-1β(IL-1β), Interleukin-2R(IL-2R), Interleukin-8(IL-8), Interleukin-10(IL-10), and Tumor Necrosis Factor (TNF-α) were measured by Chemiluminescence (Siemens, Munich, Gernamy), nevertheless, IL-6 was measured by Electrochemiluminescence(Siemens, Munich, Germany). Arterial Partial Pressure of Oxygen (PaO2) was determined by Electrochemiluminescence (Roche Diagnostics，Mannheim, Germany). Serum creatinine was assayed by Roche enzyme colorimetry and serum albumin by Roche end point colorimetry (bromocresol green) (Roche Diagnostics, Mannheim, Germany). Hemobilirubin was determined by Diazo colorimetry (Roche Diagnostics, Mannheim, Germany). Procalcitonin (PCT) was measured by Immunochomatographic assay(B.R.A.H.M.S. GmbH, Germany), and C reactive protein (CRP) by Immune scattering turbidimetry(Ward Biotechnology, Suzhou, China).

## Statistical analysis

All statistical analysis were performed using 25.0 SPSS Inc. (Chicago, IL, USA). Categorical variables were presented as percentage and analyzed using χ^2^ test between groups. The Shapiro-Wilk test, combined with the Q-Q figure was applied to test for normality of numerical variables. The data in accordance with normal distribution were presented as the mean + standard deviation (SD). The data with non-normal distribution were indicated as median (interquartile range, IQR). Comparisons between two groups following a normal distribution were analyzed using unpaired two-tailed Student’s t test. We performed the Mann-Whitney *U* test to examine differences between groups with skewed distribution. Correlation coefficients were assessed using Spearsman’s rank correlation. *P* values (two-tailed) < 0.05 were considered statistically significant. GraphPad Prism 9 software was used for mapping. For analysis, PCT levels more than 30 ng/mL were taken as 30 ng/mL. PCT levels less than 0.05 ng/mL were taken as 0.025 ng/mL. CRP less than 0.5 mg/L were taken as 0.25 mg/L. Ferritin concentrations more than 50,000 μg/L were taken as 50,000 μg/L.

## Results

The main clinical data and inflammatory biomarkers are summarized in Table 1. Sepsis subjects had longer duration of symptoms, higher SOFA score, and higher proportion admitted by Pediatric Emergency compared with gender-matched and age-matched control group. In addition, there were significant differences in respiratory rate, white blood cell count, IL-2R, IL-10, ferritin, and albumin. Compared with the controls, the sepsis individuals had higher radio of invasive ventilator therapy, longer staying in PICU, and higher mortality. Whereas, there were not significant differences in body temperature, heart rate, MAP, IL-6, IL-1β, TNF-α, IL-8, CRP, and PCT.

**Table 1:** Clinical features and laboratory indicators for children of sepsis and control

	Control	Sepsis	P
Subjects,n	323	184	
Male,n(%)	200(61.9)	115(62.5)	0.897
Age,months	18(8,39)	21.5(10.0,46.75)	0.107
Sources			< 0.001
General ward,n(%)	45(13.95)	46(25.0)	
Pediatric Emergency, n(%)	233(72.1)	**86**(**46.7**)	
Junior hospital, n(%)	45(13.95)	52(28.3)	
Duration of symptoms, days	4(2,7)	6(3,11)	< 0.001
SOFA score	0 (0,1)	2 (2,3)	< 0.001
Body temperature,°C	38.6 + 1.2	38.7 + 1.2	0.481
Respiratory rate, breaths per minute	37.3 + 12.7	42.1 + 13.4	< 0.001
Heart rate, beats per minute	144.24 + 28.15	144.55 + 29.55	0.906
MAP, mmHg	71.80 + 8.64	70.67 + 11.03	0.231
White blood cell count,10^9/L	9.62(5.95,14.70)	7.50(4.79,11.87)	0.000
IL-6,pg/mL	26.20(7.92,81.70)	33.15(8.37,119.45)	0.098
IL-1β, pg/mL	2.5(2.5,13.20)	2.5(2.5,11.95)	0.520
IL-2R,U/mL	1341.00(910.25,1968.00)	1654.50(1090.00,2486.50)	< 0.001
TNFα, pg/mL	15.20(10.60,23.00)	15.20(10.55,27.07)	0.743
IL-8, pg/mL	72.20(18.92,505.75)	71.25(24.97,354.00)	0.868
IL-10, pg/mL	11.00(6.13,23.10)	22.95(9.20,56.88)	< 0.001
Ferritin, ug/L	139.70(80.10,242.90)	236.10(107.02,777.12)	< 0.001
Albumin,g/L	40.6(38.0,43.3)	38.5(33.1,41.9)	< 0.001
C reactive protein, mg/L	13.27(0.55,60.62)	10.90(0.98,37.60)	0.454
PCT, ng/mL	0.09(0.03,0.72)	0.10(0.03,1.94)	0.11
FT3, pg/mL	**2.83** + **1.01**	**2.59** + **1.17**	0.027
FT4, ng/dl	**1.31** + 1.11	**1.15** + **0.42**	0.067
TSH, uIU/mL	1.10(0.55,2.32)	1.04(0.31,2.51)	0.064
Invasive ventilator therapy, n(%)	**3**(**9.3**)	**41**(**22.3**)	< 0.001
Length of stay in PICU, days	4.0(3.0,7.0)	7.0(4.25,10.0)	< 0.001
Mortality, n(%)	**1**(**3.1**)	**22**(**12.0**)	< 0.001

*P* values (two tails) were regarding the difference between the sepsis group and control group. Data are presented as mean + SD or median(Q25, Q75). MAP, mean arterial pressure; SOFA, Sequential Organ Failure Assessment; IL-6, Interleukin-6; IL-1β, Interleukin-1β; IL-2R, Interleukin-2R; TNF α, Tumor Necrosis Factor α; IL-8, Interleukin-8; IL-10, Interleukin-10; PCT, Procalcitonin; FT3, free triiodothyronine; FT4, free thyroxine; TSH, thyroid stimulating hormone

**Fig. 2:**
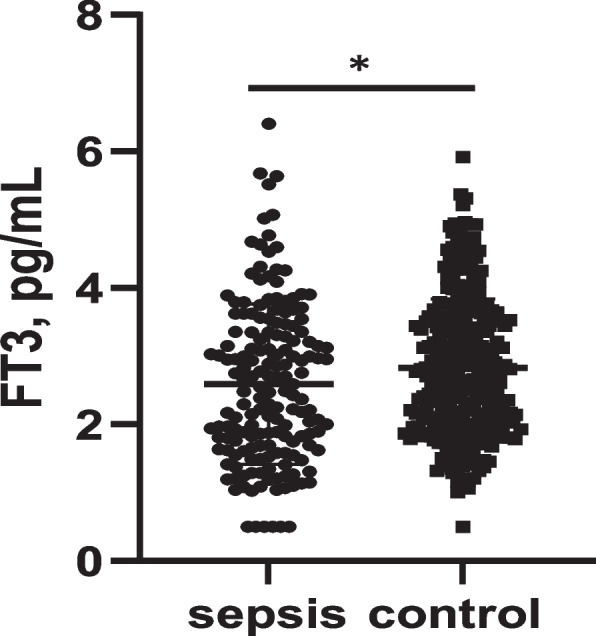
FT3 levels in sepsis and control children. Data are shown as mean + SD. * *p* < 0.05

**Fig. 3:**
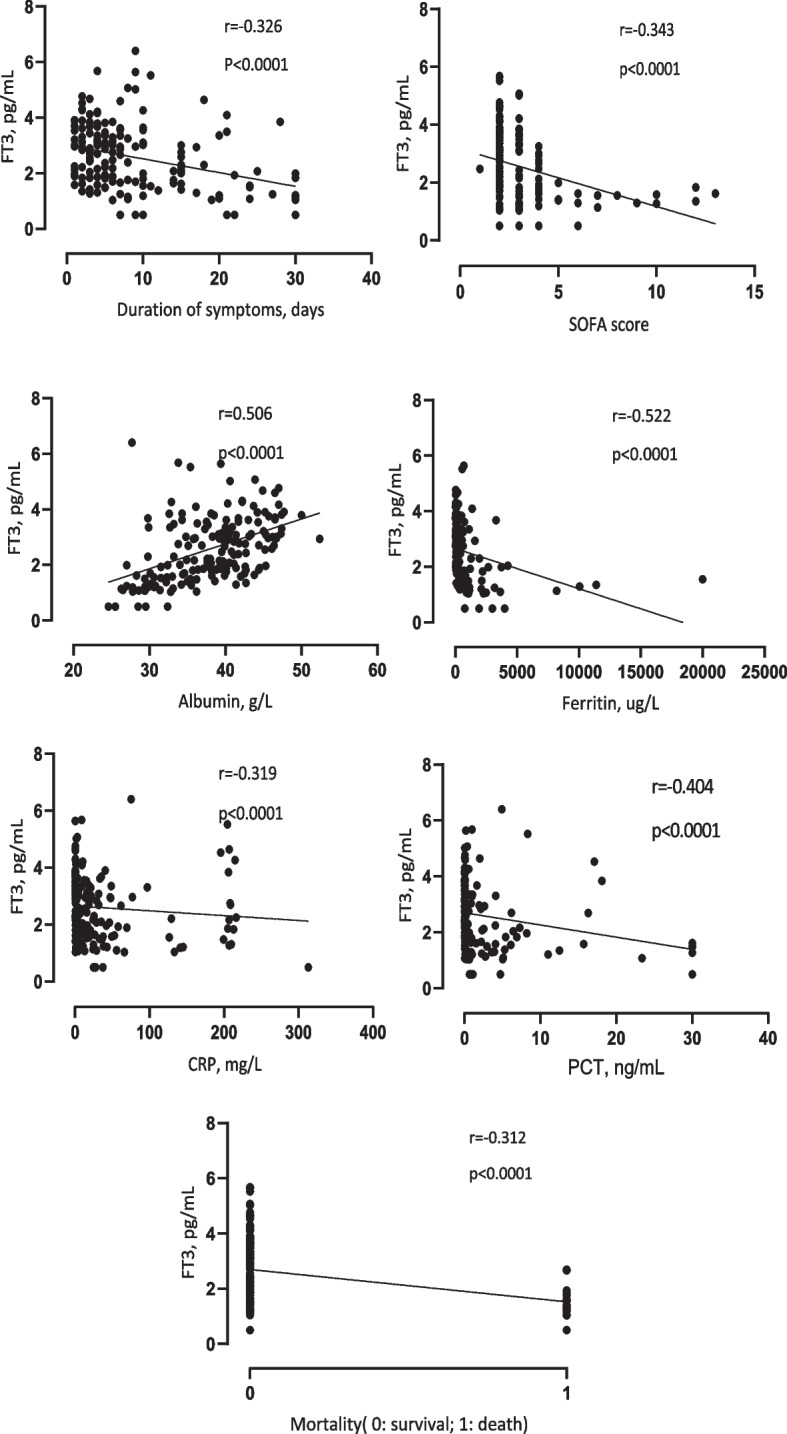
Partial correlative analysis between serum FT3 and clinical and inflammatory variables

Serum FT3 was lower in the sepsis group than in control group(2.59 + 1.17 vs 2.83 + 1.01 pg/mL, *p* < 0.05) (Fig.2). However, serum FT4 and TSH between two groups had not significant differences. Correlation analysis were performed between FT3 and clinical or inflammatory biomarkers in sepsis patients(Table 2). The analysis demonstrated that significant negative correlations of FT3 level with duration of symptoms, SOFA score, invasive ventilator therapy, mortality, CRP, PCT, ferritin, IL-6, IL-2R, and IL-10. Moreover, we observed that FT3 had the positive correlation with albumin, as well as white blood cell count(Table 2). No significant correlations were detected between FT3 and age, gender, respiratory rate, length of stay in PICU, IL-1β, IL-8, and TNF-α. The partial correlation analysis (**|**r**|** > 0.3) in sepsis patients were shown in Fig.3. To verify the correlation, linear regression analysis mentioned above were conducted (Table 3).

**Table 2:** Correlation analysis between FT3 and clinical or inflammatory biomarkers in sepsis patients

	FT3	
	r	p
Age	−0.021	0.792
Gender	−0.112	0.150
Respiratory rate	0.069	0.371
Duration of symptoms SOFA score	−0.326-0.343	< 0.0001 < 0.0001
Invasive ventilator therapy(%) Length of stay in PICU	−0.222 0.119	0.0040.124
Mortality(%)	−0.312	< 0.0001
C reactive protein	−0.319	< 0.0001
Procalcitonin	−0.404	< 0.0001
White blood cell	0.213	0.005
Albumin	0.506	< 0.0001
Ferritin	−0.522	< 0.0001
IL-6 IL-1β	−0.2040.089	0.010.302
IL-2R	−0.267	0.002
IL-8	−0.004	0.959
IL-10	−0.297	< 0.0001
TNF-α	−0.082	0.33

**Table 3:** Linear regression analysis between FT3 and partial parameters in sepsis patients

	FT3	
	β	p
Albumin	0.09	0.000
SOFA score	−0.199	0.000
Mortality	−1.171	0.000
C reactive protein	−0.002	0.231
Procalcitonin	−0.044	0.003
Duration of symptoms	−0.05	0.000
Ferritin (Ig)	−0.878	0.000

## Discussion and conclusion

We examined the thyroid hormone levels of children with sepsis and without sepsis in PICU of a single center in China, and analyzed the correlations between serum FT3 and clinical data and inflammatory biomarkers. Firstly, our study demonstrated that levels of FT3 were lower in children with sepsis compared with those with non-sepsis, consistent with the conclusions of previous researches [7–10]. Secondly, the serum FT3 was moderately correlated with multiple factors in patients with sepsis(**|**r**|** > 0.3), positive correlation with albumin, negative correlation with ferritin, CRP, PCT, duration of symptoms, SOFA score, and mortality. Then linear regression analysis was conducted to verify the correlation mentioned above，all except CRP were correlated.

The research verified that levels of FT3 in children with sepsis decreased significantly in comparison with the control group(*P* < 0.05). However, levels of FT4 and FSH did not have distinctive differences between two groups. That alteration is predominantly brought about by altered peripheral conversion of T4. Two researches before concluded that levels of FT3 and FT4 were lower in sepsis patients and animal models respectively [12, 13]. The theories explaining the role of this phenomenon include the compensatory role in relation to the oxidative stress associated with acute disease [14], and the adaptive role—as an attempt to reduce energy consumption and protect against protein catabolism [15]. In the acute phase of critical illness, such peripheral changes in thyroid hormone action and metabolism dominate. In contrast, the chronic phase of critical illness is characterized by a combination of peripheral and central adaptations [16].

Study has proved that after several days of critical illness, levels of T3 further decline [16]. In our study, levels of FT3 in sepsis children were negatively correlated with duration of symptoms at admission. The underling reason perhaps that H-P-T axe is affected more with the prolong of the course of the disease in the acute stage. SOFA score and mortality are both indicators reflecting the critical conditions of patients. FT3 levels correlated negatively with SOFA score and mortality, reflecting that low T3 could predict the severity and prognosis of patients with sepsis, which is consistent with related researches [7–10].

In our research, FT3 was positively correlated with albumin, whereas negatively correlated with ferritin. Multiple studies demonstrated that hypoalbuminemia was negatively correlated with the mortality of sepsis patients [17, 18]. Ferritin is another acute phase reactant that was mostly known for its iron storing capability than for its role in inflammation [19]. During inflammation, ferritin level increases in what has been historically considered an attempt to reduce circulating iron available for bacterial growth and development [20]. And report proved that ferrtin could be predictive indicators of children with sepsis [21].

PCT is a glycoprotein with no hormonal activity, whose sensitivity to viral and bacterial infections is high; for example, sepsis can lead to a large change in its level [22]. CRP is a non-specific and inflammation-related protein that is produced in the liver and regulated by plasma interleukin-6 (IL-6). When infection or body damage occurs, the concentration of CRP will be greatly altered [23]. In our research, the linear regression analysis verified that serum FT3 was negatively correlated with PCT, but not with CRP. The reason may be that CRP is produced limited in patients with severe sepsis which easily combined with multiple organ dysfunction, as CRP is produced in the liver.

Qinhao Li and Xiaona Gong [24] observed that PCT and CRP levels in 203 intensive care unit patients with sepsis had higher levels of PCT and CRP than non-sepsis patients. But in our observation, levels of CRP and PCT had no significant difference between sepsis children and non-sepsis children. The discrepancy of research results maybe due to the differences of the control group. The former chose individuals with colorectal cancer as control group, whereas the latter selected participants with infectious diseases as the control group, not included chronic disease.

Advantages of the research: 1) This is a clinical research from a children’s medical center in China with large sample size and complete data. 2) Sepsis 3.0 is used as the criteria for inclusion of children with sepsis. 3) Analyse the relationship between FT3 and multiple clinical or inflammatory biomarkers and conclude several moderate relative factors with FT3, which will help clinicians recognize the abnormal thyroid function through common clinical and laboratory parameters. However, there is no doubt that this study also had limitations. Firstly, it is a single center retrospective observational research, which could not be drown the causal relationship. But we conclude the correlational relationship from the clinical cases stemming from the hospital with broad sources of patients. Secondly, we perform the baseline characteristics of serum FT3, yet did not have long-term follow-up. Thereby, we are not able to predict when the abnormal serum FT3 levels of patients will return to normal and what are the potential long-term effects on the children.

In conclusion, concentrations of serum FT3 are dramatically declined in sepsis children than in control children. There are significant correlation between FT3 and multiple clinical and laboratory indicators in sepsis individuals. Our results demonstrate that recognizing the potential abnormality of thyroid function in sepsis patients and examine timely through abnormal common clinical and laboratory data is a fine option.

## Data Availability

All data and material used are included in the manuscript. The datasets used and/or analysed during the current study are available from the corresponding author upon reasonable request.

## Abbreviations

PICU: Pediatric Intensive Care Unit
GCS: Glasgow Coma Sale
MAP: Mean arterial pressure
SOFA: Sequential Organ Failure Assessment
IL-6: Interleukin-6
IL-1β: Interleukin-1β
IL-2R: Interleukin-2R
TNF α: Tumor Necrosis Factor α
IL-8: Interleukin-8
IL-10: Interleukin-10
PCT: Procalcitonin
FT3: Free triiodothyronine
FT4: Free thyroxine
TSH: Thyroid stimulating hormone.
